# Determination of a phosphorylation site in Nipah virus nucleoprotein and its involvement in virus transcription

**DOI:** 10.1099/vir.0.032342-0

**Published:** 2011-09

**Authors:** Mingshu Huang, Hiroki Sato, Kyoji Hagiwara, Akira Watanabe, Akihiro Sugai, Fusako Ikeda, Hiroko Kozuka-Hata, Masaaki Oyama, Misako Yoneda, Chieko Kai

**Affiliations:** 1 Laboratory Animal Research Center, Institute of Medical Science, University of Tokyo, 4-6-1 Shirokanedai, Minato-ku, Tokyo 108-8639, Japan; 2 International Research Center for Infectious Diseases, Institute of Medical Science, University of Tokyo, 4-6-1 Shirokanedai, Minato-ku, Tokyo 108-8639, Japan; 3 Medical Proteomics Laboratory, Institute of Medical Science, University of Tokyo, 4-6-1 Shirokanedai, Minato-ku, Tokyo 108-8639, Japan

## Abstract

Many viruses use their host’s cellular machinery to regulate the functions of viral proteins. The phosphorylation of viral proteins is known to play a role in genome transcription and replication in paramyxoviruses. The paramyxovirus nucleoprotein (N), the most abundant protein in infected cells, is a component of the N–RNA complex and supports the transcription and replication of virus mRNA and genomic RNA. Recently, we reported that the phosphorylation of measles virus N is involved in the regulation of viral RNA synthesis. In this study, we report a rapid turnover of phosphorylation in the Nipah virus N (NiV-N). The phosphorylated NiV-N was hardly detectable in steady-state cells, but was detected after inhibition of cellular protein phosphatases. We identified a phosphorylated serine residue at Ser451 of NiV-N by peptide mass fingerprinting by electrospray ionization–quadrupole time-of-flight mass spectrometry. In the NiV minigenome assay, using luciferase as a reporter gene, the substitution of Ser451 for alanine in NiV-N resulted in a reduction in luciferase activity of approximately 45 % compared with the wild-type protein. Furthermore, the substitution of Ser451 for glutamic acid, which mimics a phosphoserine, led to a more significant decrease in luciferase activity – approximately 81 %. Northern blot analysis showed that both virus transcription and replication were reduced by these mutations. These results suggest that a rapid turnover of the phosphorylation of NiV-N plays an important role in virus transcription and replication.

## Introduction

Nipah virus (NiV) is a recently emerged zoonotic virus that causes encephalitic and respiratory illness in humans and livestock, with a high mortality rate (40–70 %) in humans (Chua *et al.*, 2000; WHO, 2004). Since its identification in 1998–1999 in Malaysia, NiV has been associated with several outbreaks of human fetal viral encephalitis in South Asia (Chua *et al.*, 2000) and there is increasing evidence of person-to-person transmissions (Hsu *et al.*, 2004; WHO, 2004).

NiV is a negative-stranded, ssRNA virus that belongs to the genus *Henipavirus* in the family *Paramyxoviridae* (Mayo, 2002a, b) and is composed of six structural proteins: nucleoprotein (N), phosphoprotein (P), matrix protein (M), fusion protein (F), glycoprotein (G) and large protein (L). The N protein encapsidates the genomic RNA and forms a nucleocapsid. This serves as a template for virus replication and transcription, which are catalysed by an RNA-dependent RNA polymerase (RdRp) that is composed of P and L proteins (Wang *et al.*, 2001).

Paramyxovirus N proteins possess a highly conserved structure. The N-terminal 80 % of the protein forms a globular body, whereas the C-terminal 20 % appears to be a tail extending from the N-terminal body (Lamb & Parks, 2006). The genus *Henipavirus* is related closely to the genus *Morbillivirus* within the family *Paramyxoviridae*. Measles virus (MV), which belongs to the genus *Morbillivirus*, has a well-characterized N protein. MV-N is also divided into two regions: (i) an N-terminal domain, N-CORE, which has a well-conserved sequence (aa 1–400), and (ii) a hypervariable C-terminal domain, N-TAIL (aa 401–525) (Heggeness *et al.*, 1980, 1981; Longhi *et al.*, 2003). MV-N-CORE contains all of the necessary components for self-assembly and RNA binding, as N proteins that are composed only of the core region can encapsidate newly synthesized RNA into nucleocapsid-like particles. MV-N-TAIL contains the region responsible for N–P binding and is consequently required for virus transcription (Longhi *et al.*, 2003).

NiV-N is composed of 532 aa, slightly larger than MV-N (Wang *et al.*, 2001). A recent study revealed that deletion of ≤128 aa from the C terminus of NiV-N does not impair the formation of the NiV-N herringbone-like nucleocapsid structure (Ong *et al.*, 2009). However, deletion of 129 aa or more from the C terminus completely abolishes the formation of the herringbone-like particles and the protein aggregates into spherical particles (Ong *et al.*, 2009). Furthermore, one of the binding sites of the P protein was mapped to the 29 aa (aa 468–496) C-terminal region of NiV-N (Chan *et al.*, 2004). It is assumed that NiV-N has a similar function to MV-N.

It has been reported that MV-N has five phosphorylated serine and threonine residues (Gombart *et al.*, 1995; Segev *et al.*, 1995). Recently, we identified the two major phosphorylation sites of MV-N by matrix-assisted laser desorption/ionization–tandem time-of-flight (MALDI-TOF/TOF) and electrospray ionization–quadrupole time-of-flight (ESI-Q-TOF) mass spectrometry analyses (Hagiwara *et al.*, 2008). Both phosphorylation sites were in the C-terminal region, where phosphorylation was required for efficient virus transcription in the MV minigenome assay system.

In the present study, we determined that NiV-N is also phosphorylated. However, there is a rapid turnover of phosphorylation, which is detectable only after inhibition of cellular protein phosphatases. In addition, we identified the phosphorylation site as Ser451 and examined the role of phosphorylation of NiV-N in the transcription and replication of virus RNA.

## Results

### NiV-N is dephosphorylated immediately by cellular protein phosphatases

**Fig. 1.**
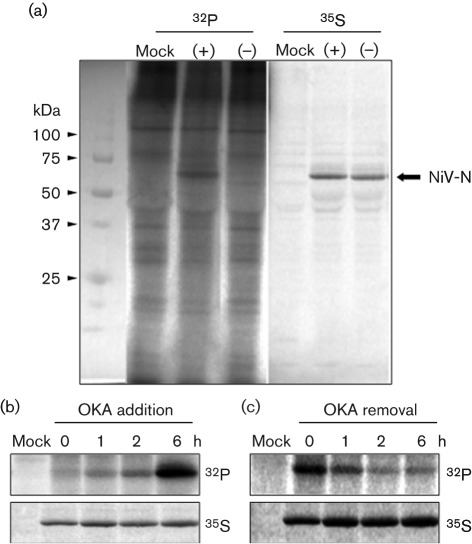
Phosphorylation of NiV-N after OKA treatment. (a) NiV-N protein was expressed in COS-7 cells and labelled with ^32^P or ^35^S in the presence (+) or absence (−) of OKA. (b) Time-dependent experiments of phosphorylation of NiV-N. In the presence of ^32^P or ^35^S, 100 nM OKA was added to the culture medium. Cells were harvested at several time points. (c) After exposure to OKA for 24 h in the presence of ^32^P or ^35^S, OKA was removed from the culture medium and cells were harvested at several time points.

To examine whether NiV-N is phosphorylated in host cells, NiV-N was expressed transiently in COS-7 cells in the presence of ^32^P-labelled phosphate. After cell lysis, NiV-N was immunoprecipitated with antiserum against NiV-N, and was subjected to SDS-PAGE and autoradiography. However, ^32^P-labelled NiV-N was not detected (Fig. 1a). Recently, it has been reported that phosphorylation of the P protein at Ser54 of the human respiratory syncytial virus (HRSV), another member of the family *Paramyxoviridae*, is detected only by inhibition of cellular protein phosphatases, due to its immediate dephosphorylation (Asenjo *et al.*, 2005). Therefore, to confirm whether NiV-N is also dephosphorylated immediately, ^32^P-labelling experiments were performed in the presence of okadaic acid (OKA), an inhibitor of cellular protein phosphatases. In the presence of OKA, strong ^32^P labelling of NiV-N was observed (Fig. 1a). To investigate the kinetics of phosphorylation and dephosphorylation of NiV-N, time-dependent experiments were performed. The amount of ^32^P-labelled NiV-N increased gradually up to 6 h after OKA administration (Fig. 1b), and decreased within 2 h after OKA removal (Fig. 1c). These results indicate that NiV-N has a rapid turnover of phosphorylated residues and that host cellular activity is involved in regulating the phosphorylation of NiV-N.

### NiV-N is phosphorylated at Ser451

**Fig. 2.**
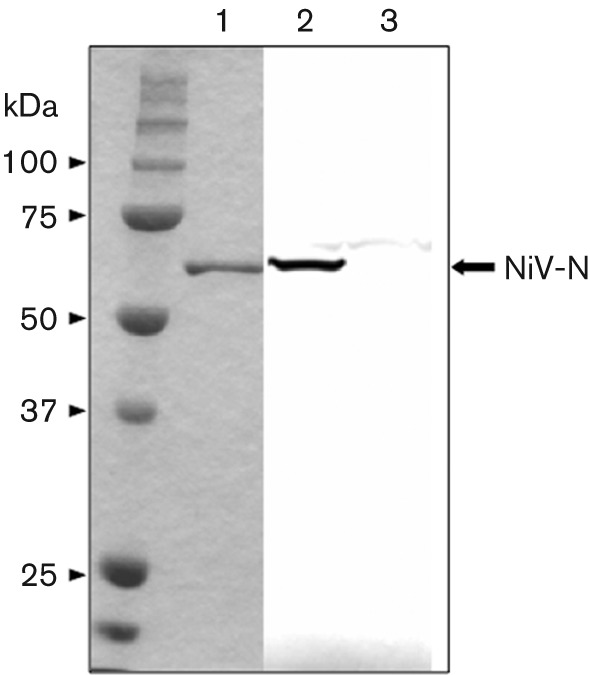
SDS-PAGE and Western blotting of purified NiV-N. Recombinant NiV-N was expressed in COS-7 cells and purified by caesium chloride density-gradient centrifugation. Samples were separated by SDS-PAGE (10 % gel) and detected by CBB staining (lane 1). Purified NiV-N was detected by Western blotting with NiV-N-specific antibody (lane 2). An empty vector plasmid was also transfected into the cells and the lysate was utilized as a negative control (lane 3).

**Fig. 3.**
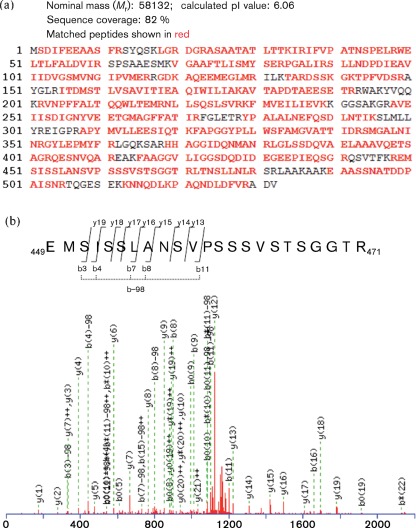
ESI-Q-TOF MS of NiV-N. (a) Digested peptides were analysed by ESI-Q-TOF MS, followed by a database search using Mascot ver. 2.0 (Matrix Science). Identified peptides are shown in red. (b) The spectrum was interpreted using the software DataExplore. The sequences from the N and C termini, and the position of the modified residues, were determined based on b and y ions, respectively. A 98 Da neutral loss of ions is shown above the sequences as b−98.

To identify the phosphorylation sites for NiV-N, NiV-N was expressed transiently in COS-7 cells and the nucleocapsid complex was purified by caesium chloride density-gradient centrifugation (Hagiwara *et al.*, 2008; Oglesbee *et al.*, 1989). The obtained fraction was analysed by Coomassie brilliant blue (CBB) staining (Fig. 2, lane 1) and Western blotting using an antiserum against NiV-N (Fig. 2, lane 2), which showed that NiV-N was purified homogeneously. The purified NiV-N was trypsinized by in-gel digestion, and the resultant peptides were analysed by ESI-Q-TOF MS, followed by a database search with Mascot ver. 2.0 (Matrix Science). The total sequence coverage was 82 % (Fig. 3a). In the MS/MS spectrum of the precursor ion that corresponds to the peptide 449-EMSISSLANSVPSSSVSTSGGTR-471, five fragment ions (b3, b4, b7, b8 and b11) were accompanied by their dephosphorylated ions (b3−98, b4−98, b7−98, b8−98 and b11−98, respectively), whereas seven fragment ions (y13, y14, y15, y16, y17, y18 and y19) were not. This spectrum indicated that Ser451 is a phosphorylation site on NiV-N (Fig. 3b).

**Fig. 4.**
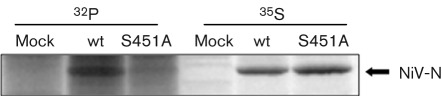
Radioisotope labelling and immunoprecipitation of wt and mutant N proteins. Wt N protein and mutants were expressed in COS-7 cells and labelled with ^32^P or ^35^S in the presence of OKA. After immunoprecipitation with NiV-N-specific antibody, samples were resolved by SDS-PAGE (10 % gel) and detected with an FLA-5100 imaging system (Fujifilm).

To confirm the predicted phosphorylation site, Ser451 in NiV-N was substituted with alanine (A), which is unable to be phosphorylated, and the resultant NiV-N mutant, NiV-N S451A, was expressed in COS-7 cells in the presence of ^32^P and OKA. The S451A mutation greatly reduced the phosphorylation of NiV-N (Fig. 4), which indicated that Ser451 is the major phosphorylation site on NiV-N. Ser451 is located in the C-terminal region, as in MV-N.

### NiV-N Ser451 is involved in efficient transcription and replication of the NiV minigenome

NiV-N is a major component of the virus ribonucleoprotein (RNP) complex and is required for virus mRNA transcription and genome replication. We reported previously that the phosphorylation of MV-N upregulates the transcriptional activity of MV minigenomic RNA (Hagiwara *et al.*, 2008). Therefore, to examine the role of rapid-turnover phosphorylation of NiV-N Ser451 in virus transcription and/or replication, we developed an NiV minigenome assay using firefly luciferase as a reporter gene, flanked by the leader and trailer sequences of the NiV genome. In addition to the NiV-N S451A mutant, we constructed another mutant NiV-N, S451E, in which S451 was substituted with glutamic acid (E) as a phosphoserine mimic (Wu *et al.*, 2002).

**Fig. 5.**
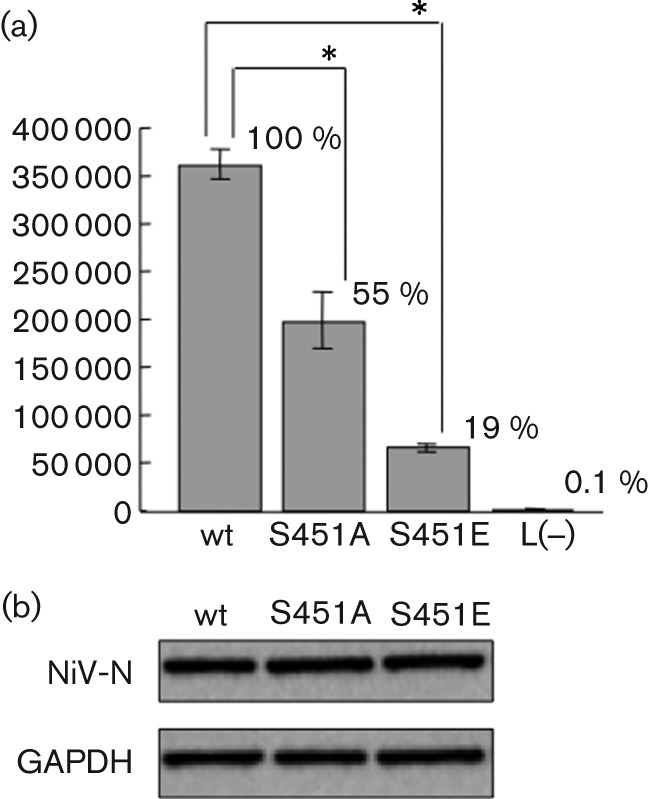
Minigenome assay. (a) Negative-strand minigenomic RNA containing the ORF of the luciferase gene was transfected into HEK 293 cells expressing wt or mutant N protein, together with P and L proteins. Luciferase activity of cell extracts was measured using a Mini Lumat LB 9506 (Berthold Technologies). The data represent means±sd for triplicate samples. Statistical analysis was performed using Student’s *t*-test; *P<0.05 compared with NiV-N-wt. (b) Protein expression was detected by Western blotting with NiV-N-specific antibody and glyceraldehyde-3-phosphate dehydrogenase (GAPDH) antibody.

HEK 293 cells were transfected with the negative-strand minigenomic RNA and supporting plasmids that expressed NiV-N [wild-type (wt), S451A or S451E], -P and -L. A luciferase assay was performed at 24 h post-transfection. Western blotting of cell lysates from each minigenome assay showed that there was no significant difference in the expression of NiV-N between wt, S451A and S451E (Fig. 5b). However, the luciferase activity in NiV-N S451A was 45 % lower than that of wt NiV-N (Fig. 5a), which suggests that phosphorylation at Ser451 is required for efficient transcription and/or replication of virus RNA. The luciferase activity in NiV-N S451E, which was a mimic of phosphoserine, was 81 % lower than that in wt NiV-N (Fig. 5a). Therefore, it can be assumed that retaining a state of phosphorylation is undesirable for NiV, because it suppresses replication and/or transcription.

**Fig. 6.**
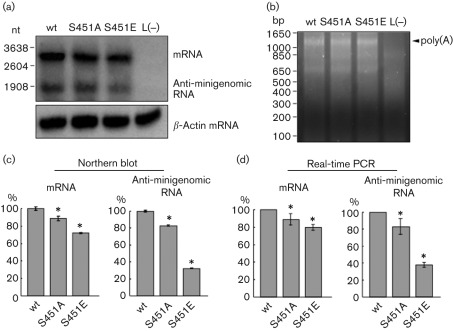
(a) Detection of minigenomic transcript and anti-minigenomic RNA with an antisense luciferase (Luc) probe. Total RNA was prepared from HEK 293 cells by minigenome assay with NiV-N-wt, NiV-N-S451A and NiV-N-S451E, and was hybridized with the antisense Luc probe. The respective anti-minigenomic RNA and mRNA are indicated. Total RNA was also hybridized with a β-actin probe. (b) Confirmation of the length of the poly(A) tail of the luciferase mRNA in the minigenome assay. Total RNA (described above) was subjected to 3′ RACE as described in Methods, and amplified cDNA of the poly(A) tail was separated in agarose gel. (c) Quantitative analysis of (a). The amounts of minigenomic transcripts and anti-minigenomic RNA products in relation to those for wt were quantified as described in Methods. (c) Relative quantification of mRNA and anti-minigenomic RNA by semi-quantitative real-time PCR. Total RNA prepared from the minigenome assay was reverse-transcribed with oligo(dT) primer for mRNA or with a primer specific for NiV trailer sequence. The cDNA was subjected to real-time PCR, as described in Methods. For normalization, GAPDH mRNA was used as an internal standard. These data represent means±sd for triplicate samples. Statistical analysis was performed using Student’s *t*-test; *P<0.05 compared with NiV-N-wt.

To investigate the effect of NiV-N phosphorylation on replication and transcription using the NiV minigenome assay, the expression of luciferase mRNA and positive-strand anti-minigenomic RNA was quantified by Northern blotting. Minigenome assays were performed under the same conditions as described above and total RNA was extracted. Luciferase mRNA and positive-strand anti-minigenomic RNA were detected with an antisense riboprobe located within the luciferase gene. Luciferase mRNA appeared as the upper band, apparently >1000 nt longer than the antigenomic RNA (1866 nt) (Fig. 6a). We determined the length of the poly(A) tail in the luciferase mRNA by 3′ RACE; approximately 1200 nt poly(A) tail was detected (Fig. 6b). Thus we performed quantitative analysis of the luciferase mRNA and antigenomic RNA by Northern blotting (Fig. 6c). For the S451A mutation, the levels of anti-minigenomic RNA and mRNA were reduced slightly compared with those for wt NiV-N. For the S451E mutation, the level of mRNA was 26 % lower than that for wt, whereas the level of anti-minigenomic RNA decreased by 68 %. We further performed real-time PCR analysis to detect mRNA and anti-minigenomic RNA levels; it showed a similar pattern to Northern blotting (Fig. 6d), as reported previously (Sleeman *et al.*, 2008). These results suggest that replication was affected more than transcription.

### Mutation of Ser451 of NiV-N does not affect nucleocapsid or N–P complex formation

**Fig. 7.**
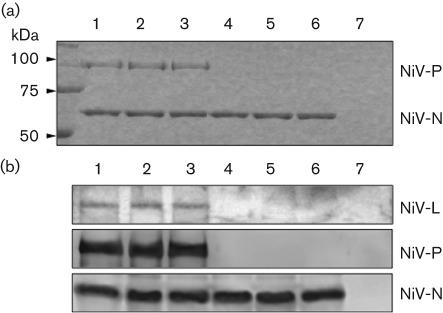
Nucleocapsid and N–P–L complex formation assay. NiV-N-wt (lane 4), NiV-N-S451A (lane 5), NiV-N-S451E (lane 6) and NiV-P (lane 7) were expressed individually in COS-7 cells and subjected to caesium chloride density-gradient centrifugation. Alternatively, cell extracts of NiV-P and NiV-L were mixed with NiV-N-wt (lane 1), NiV-N-S451A (lane 2) or NiV-N-S451E (lane 3), and the N–P–L complexes were purified by centrifugation in the same manner. Each sample was resolved by SDS-PAGE and detected by (a) CBB staining or (b) Western blotting using NiV-N-, -P- and -L-specific antibodies, respectively.

It has been reported that the MV-N-TAIL region binds to the P protein, a component of the RdRp composed of P and L proteins, and thus supports virus transcription and replication (Bankamp *et al.*, 1996; Johansson *et al.*, 2003; Liston *et al.*, 1997; Longhi *et al.*, 2003). In our own studies of MV-N phosphorylation, the loss of phosphorylation of MV-N did not affect its binding to the P protein (Hagiwara *et al.*, 2008). To determine whether the reduction in luciferase activity observed with the mutant proteins was caused by the abrogation of nucleocapsid formation and/or the loss of binding to RdRp, we first investigated the efficiency of nucleocapsid formation of the mutants. NiV-N-wt, S451A and S451E were expressed in cells and then subjected to caesium chloride centrifugation. As shown in Fig. 7, S451A and S451E formed nucleocapsid at a rate equal to wt (lanes 4–6). Next, NiV-N-wt, S451A and S451E were mixed with NiV-P and -L, and purified by caesium chloride centrifugation. If they bound to NiV-N, they were purified together as a nucleocapsid complex. There was no significant difference observed in the amount of NiV-N and co-purified NiV-P and -L among wt, S451A and S451E (Fig. 7, lanes 1–3). Solely expressed NiV-P (Fig. 7, lane 7) and -L (data not shown) were not detected in the fraction. In addition, we confirmed that S451A and S451E formed soluble N–P complex, similarly to N-wt, by using a co-immunoprecipitation assay (data not shown). These results indicate that mutation of Ser451 of NiV-N does not affect either nucleocapsid or N–P–L complex formation.

## Discussion

Many viruses have evolved mechanisms to use cellular machinery to modify their proteins and regulate their functions. Recently, we have identified the phosphorylation sites on the MV-N protein and revealed that phosphorylation is required for efficient transcription of minigenomic RNA (Hagiwara *et al.*, 2008). Phosphorylation of the N protein has also been reported in Sendai virus (Hsu & Kingsbury, 1982) and mumps virus (Naruse *et al.*, 1981) of the family *Paramyxoviridae*. Likewise, the N proteins of rabies virus (RV) (Wu *et al.*, 2002), Marburg virus (MBGV) (Becker *et al.*, 1994) and Ebola virus (Elliott *et al.*, 1985), which belong to the order *Mononegavirales*, have also been reported to undergo phosphorylation. In the case of RV, it has been reported that both virus transcription and replication are reduced when the RV-N protein is not phosphorylated (Wu *et al.*, 2002). In the case of MBGV, it has been reported that only the phosphorylated N protein can form nucleocapsid complexes and interact with the genomic RNA (Becker *et al.*, 1994). Thus, phosphorylation of the virus N protein is a very important step in the life cycle of the virus.

In the present study, we revealed that NiV-N also undergoes phosphorylation at Ser451 (Fig. 3). However, the phosphorylation state of the NiV-N protein is different from that of MV-N, phosphorylation of which is stably detected in cells (Hagiwara *et al.*, 2008). However, we also found that phosphorylation of NiV-N was not stable, but showed rapid turnover (Fig. 1), suggesting that NiV-N phosphorylation/dephosphorylation is regulated by a cellular kinase–phosphatase system during the NiV life cycle.

Hendra virus (HeV) is also a member of the genus *Henipavirus*. Identity at the amino acid level between the N proteins of HeV and NiV is 92.1 % (Wang *et al.*, 2001). Interestingly, HeV-N also possesses a Ser at aa 451, suggesting that HeV-N also undergoes phosphorylation. Further study on HeV-N protein phosphorylation should be carried out.

We have ascertained previously that MV-N is phosphorylated in the tail region, at Ser479 and Ser510 (Hagiwara *et al.*, 2008). The tail region of MV-N possesses three conserved regions that are enriched in hydrophobic residues: box-1, box-2 and box-3, which correspond to MV-N aa 401–420, 489–506 and 517–525, respectively (Zhang *et al.*, 2005). Thus, the phosphorylation sites of MV-N are located in the neighbourhood of box-2, which contains the binding site for the MV-P protein X domain, where the P protein tethers the viral polymerase to the nucleocapsid in support of transcription and genome replication (Zhang *et al.*, 2005). Our previous study indicated that phosphorylation of MV-N does not influence its binding to MV-P, but instead upregulates the transcriptional activity of minigenomic RNA (Hagiwara *et al.*, 2008). In the case of NiV-N, it has been shown that the NiV-N C-terminal region contains a P-binding domain at aa 468–496 (Chan *et al.*, 2004). Therefore, the phosphorylation site of NiV-N that we identified in this study, Ser451, is also located close to the P-binding region, similar to that of MV-N. As shown in Fig. 7, phosphorylation-deficient NiV-N bound P to the same extent as the wt NiV-N protein. Thus, phosphorylation might not influence the direct interaction between NiV-N and -P, as is the case for MV.

To clarify the role of phosphorylation of NiV-N, we utilized the NiV minigenome assay system. We observed that virus transcription and replication were reduced when NiV-N was not phosphorylated in the minigenome system (Fig. 5). In the case of RV, the N protein contains one phosphorylation site, and virus transcription and replication are also reduced in this assay when using the minigenome and a recombinant virus with the phosphorylation site substituted with alanine (Wu *et al.*, 2002). Interestingly, the phosphoserine mimic of the RV-N protein shows equivalent minigenome activity and growth rate of the recombinant virus to that with wt RV-N protein. Similarly, our previous study using an MV minigenome assay demonstrated that when MV-N is not phosphorylated, luciferase activity is reduced compared with phosphorylated N (Hagiwara *et al.*, 2008), whilst the phosphoserine mimic of MV-N restores the activity to some extent (unpublished data). In contrast, in the case of NiV-N, the mutant S451E, which has a similar negative charge to NiV-N, shows lower luciferase activity than the S451A mutant (Fig. 5). This suggests that the short duration of phosphorylation of NiV-N is important for optimal transcription/replication activity. These results are consistent with the observation that phosphorylated NiV-N was hardly detected in steady-state cells (Fig. 1a). In particular, Northern blot analysis and real-time PCR showed that the replication efficiency decreased in S451E, whilst replication and transcription decreased to a similar level in S451A (Fig. 6). These results imply that the rapid dephosphorylation of NiV-N enhances mainly this replication step. It remains a possibility that the E mutation itself caused the decrease in replication, but the S451E mutant retained both nucleocapsid-formation and P- and L-binding activities (Fig. 7). Thus, it suggests strongly that the replication-specific downregulation by S451E was not caused by the mere introduction of the mutation, but rather the constitutive negative charge on the S451 residue. In paramyxoviruses, it is believed that the RdRp travels from upstream to downstream on the nucleocapsid, which encapsulates the genomic RNA during transcription and replication, and that the efficient transfer of P protein between adjacent N proteins correlates with the efficiency of virus RNA synthesis (Zhang *et al.*, 2005). The phosphorylation of NiV-N S451 introduces a negative charge to the site. Thus, rapid turnover of phosphorylation at S451 might bring about change in the conformation and/or activities of the N protein and the ribonucleocapsid–RNA polymerase complex. The results of this study shed light on the mechanism of the regulation of virus RNA synthesis of NiV, which nonetheless remains somewhat unclear.

Previous studies have revealed that the HRSV-P protein possesses a rapid-turnover phosphorylation site at Ser54, and that the phosphorylation is not involved in the formation of the P–N or P–L complexes (Asenjo *et al.*, 2005). Recently, using a recombinant HRSV, Asenjo *et al.* (2008) demonstrated that the rapid-turnover phosphorylation of the HRSV-P protein is required for the virus uncoating step in the early stage of infection, during which the virus RNP particle is liberated from the M protein and the virus proceeds to a rapid growth cycle. In the case of NiV-N, we found that rapid turnover of N-protein phosphorylation at S451 is not involved in N–P complex formation *in vitro* (Fig. 7), but is instead implicated in efficient virus genome replication and transcription in the NiV minigenome assay (Fig. 6). However, additional function(s) of the phosphorylation of the N protein, important for the life cycle of NiV *in vivo*, may exist. In a previous study, we established an NiV reverse-genetics system (Yoneda *et al.*, 2006). Using this system, rescued NiV was shown to be similar to wt virus and to retain severe pathogenicity in an animal model by experimental infection (Yoneda *et al.*, 2006). This system will be used for a future study that will aim to elucidate the mechanism of rapid-turnover phosphorylation of NiV-N and its role in the virus growth cycle.

## Methods

### 

#### Cells.

COS-7 (monkey kidney) and HEK 293 (human kidney) cells were maintained in Dulbecco’s modified Eagle’s medium (DMEM; Sigma) containing 5 % FBS (Sigma-Aldrich), 100 U penicillin G ml^−1^ and 100 µg streptomycin ml^−1^ (Invitrogen).

#### Construction of plasmids.

The N, P and L gene ORFs were amplified by PCR using KOD Plus ver. 2 (Toyobo) primers, including *Sal*I/*Bam*HI (N), *Mlu*I/*Bsm*I (P) and *Mlu*I*/Not*I (L) sites, and an NiV-supporting plasmid that encodes the NiV-N, -P and -L genes as a template (Radecke *et al.*, 1995; Yoneda *et al.*, 2006). Amplified PCR products were purified by using a MinElute PCR Purification kit (Qiagen), inserted between *Sal*I/*Bam*HI (N), *Mlu*I/*Bsm*I (P) and *Mlu*I*/Not*I (L) sites in the pCAGGS mammalian expression vector (Tokui *et al.*, 1997) and designated pCAGGS-NiV-N, -P and -L, respectively. Mutant NiV-N plasmids S451A and S451E were generated by mutagenesis using *Pfu* DNA polymerase (Stratagene) according to the manufacturer’s instructions.

#### Mammalian expression of NiV-N and purification of the nucleocapsid.

COS-7 cells (1.0×10^6^) were transfected with 6.0 µg pCAGGS-NiV-N by using FuGene 6 transfection reagent (Roche), according to the manufacturer’s instructions. Forty-eight hours after transfection, cells were lysed with 500 µl lysis buffer that contained 10 mM Tris/HCl (pH 7.8), 150 mM NaCl, 1 mM EDTA, 1 % NP-40, 1 mM Na_3_VO_4_, 50 mM NaF and protease inhibitor cocktail (BD Biosciences) at 4 °C for 30 min. The lysate was centrifuged at 20 400 ***g*** for 10 min and the supernatant was layered onto a discontinuous gradient that contained 25, 30 and 40 % (w/w) caesium chloride prepared in lysis buffer without NP-40. After centrifugation at 55 000 r.p.m. for 30 min with a SW55Ti rotor (Beckman), the band material that contained NiV-N was collected. Finally, NiV-N was pelleted by ultracentrifugation at 70 000 r.p.m. for 10 min with a TLA100.3 rotor (Beckman) and suspended in 20 µl PBS.

#### Production of NiV-N-specific antibody.

Antiserum against NiV-N was raised in a rabbit by immunization with approximately 120 µg purified N protein mixed with complete Freund’s adjuvant (Difco). Serum was collected 6 weeks after injection. The animal experiment was approved by the Animal Ethics Committee in our institute and conducted according to the institutional guidelines for animal experiments.

#### SDS-PAGE and Western blotting.

Purified proteins were separated by SDS-PAGE (10 % gel) under reducing conditions and stained with CBB. Western blotting was performed according to a standard method. In brief, after separation by SDS-PAGE, proteins were transferred to an Immobilon-P membrane (Millipore) and incubated with a 1000-fold dilution of rabbit antiserum against NiV-N. The membrane was then incubated with a 1000-fold dilution of HRP-conjugated anti-rabbit IgG antibody (DakoCytomation). Detection was carried out using ECL Western blotting Detection Reagents (Amersham Biosciences) and the LAS-1000UV mini system (Fujifilm).

#### Detection of phosphorylated NiV-N by autoradiography after OKA treatment.

COS-7 cells (1.5×10^5^) were transfected with 1.6 µg pCAGGS-NiV-N plasmid for wt or phosphorylation-site mutants, as described above. Twenty-four hours after transfection, ^32^P (PerkinElmer) or ^35^S (EXPRESS ^35^S Protein Labeling Mix; PerkinElmer) and 100 nM OKA (Calbiochem) were added to the medium and cultured for 6–24 h. The cells were lysed in lysis buffer that contained 5 mM EDTA, 0.5 % Triton X-100, 1 mM Na_3_VO_4_, 50 mM NaF and protease inhibitor cocktail in PBS at 4 °C for 1 h, followed by clarification by centrifugation at 20 400 ***g*** for 30 min. Rabbit antiserum against NiV-N protein and protein–G Sepharose 4 Fast Flow (GE Healthcare) were added to the supernatant and incubated at 4 °C for 16 h with gentle rotation. Afterwards, immunoprecipitation samples were resolved by SDS-PAGE. The amount of labelled N protein was estimated by using the FLA-5100 imaging system (Fujifilm).

#### In-gel digestion of N protein by trypsin.

Purified NiV-N was subjected to SDS-PAGE and stained with a SeePico CBB Stain kit (Benebiosis). A single band of approximately 58 kDa was excised from the gel and then destained five times with 50 mM NH_4_HCO_3_ in 50 % methanol. After drying the gel in 100 % CH_3_CN, 2.5 pmol trypsin (Trypsin Gold Mass Spectrometry Grade; Promega) in 50 µl, 10 mM Tris/HCl (pH 8.5) was added and in-gel digestion was carried out at 37 °C for 16 h. Digested peptides were extracted by treating the gel twice with 0.1 % trifluoroacetic acid (TFA) in 50 % CH_3_CN, and finally extracted once more by 0.1 % TFA in 80 % CH_3_CN for complete extraction.

#### ESI-Q-TOF MS.

Digested peptides were applied to a high-resolution nanoflow reversed-phase capillary LC (DiNa; KYA Technologies) coupled with an electrospray source and Q-TOF tandem mass spectrometer (Q-Tof-2; Micromass Ltd), as described previously (Oyama *et al.*, 2004, 2007). The acquired MS/MS mass spectral data were processed using Mascot ver. 2.0 (Matrix Science) against each database, with maximum tolerance of 100 p.p.m. in MS data and 0.15 Da in MS/MS data. The candidate peptide sequences were screened using probability-based mowse scores that exceeded their thresholds (*P*<0.05).

#### Minigenome assay.

The NiV minigenome encoding luciferase was constructed essentially as described by Halpin *et al.* (2004), with the modification that the firefly luciferase gene was used instead of the chloramphenicol acetyltransferase gene. The resulting minigenome fragment was sequenced and cloned into *Eag*I–*Bsm*I sites of pMDB1 (Baron & Barrett, 1997). *In vitro* transcription was performed using T7 RiboMAX Large-Scale RNA Production systems (Promega), according to the manufacturer’s instructions, and negative-strand minigenomic RNA was purified using MicroSpin G50 columns (Amersham Biosciences).

HEK 293 cell monolayers in 24-well plates, grown to 90 % confluence, were infected with recombinant vaccinia virus vTF7-3 at an m.o.i. of 3. After 30 min infection, cells were washed with PBS and transfected with NiV minigenome luciferase RNA (150 ng) that carried the luciferase reporter gene, together with supporting plasmids that carried N (312.5 ng per well), P (200 ng µg^−1^) and L (100 ng per well) (Halpin *et al.*, 2004; Yoneda *et al.*, 2006), using DMRIE-C reagent (Invitrogen) according to the manufacturer’s protocol. After 4 h transfection, medium was removed. Cells were washed with OPTI-MEM (Invitrogen GIBCO) and incubated with DMEM with 5 % FBS for 20 h. Cells were lysed and subjected to the reporter assay using Pickagene reagent (Toyo) and a Mini Lumat LB 9506 luminometer (Berthold Technologies), according to the manufacturers’ instructions.

#### Preparation of a minigenome-specific RNA probe.

A PCR fragment of luciferase sequences from nucleotide positions 521 to 901 was amplified by Phusion High-Fidelity DNA Polymerase (Finnzymes). The T7 promoter sequence was also appended to the reverse primer for RNA transcription *in vitro*. The PCR product was extracted by the Wizard SV Gel and PCR Clean-Up system (Promega) after agarose gel electrophoresis. DNA was extracted by phenol/chloroform/isoamylalcohol extraction. The purified template DNA was transcribed *in vitro* using T7 RNA polymerase by a DIG RNA Labelling kit (Roche Applied Science), according to the manufacturer’s instructions.

#### Northern blot analysis of minigenomic RNA synthesis.

Total RNA was extracted after transfection for the minigenome assay with ISOGEN (Nippon Gene), following the manufacturer’s instructions. RNA was extracted and separated on a 1.5 % MOPS–formaldehyde agarose gel at 100 V for 90 min at 4 °C, and transferred onto a Hybond-N(+) nitrocellulose membrane (GE Healthcare). Hybridization of the membrane with a minigenome-specific RNA probe was performed at 68 °C for 18 h. Signals from the hybridized probes were detected by using a DIG DNA/RNA Labelling and Detection kit (Roche Applied Science) and CDP-Star detection reagent (GE Healthcare), according to the manufacturers’ instructions, and visualized by exposure to LAS-1000 mini (Fujifilm). Each band was quantified by software from Science Lab 2001 Image Gauge (Fujifilm) and the density of each band was compensated with its background, which contains an equivalent area from this lane with no signals. The value of density for the wt samples was set as 100 %, and the relative density of mutant samples was calculated by dividing the density value of mutant samples by that of the controls.

#### Measurement of poly(A) tail length.

Total RNA extracted from the minigenome assay described above was subjected to 3′ RACE for determination of the length of the poly(A) tail in the luciferase mRNA using an ALL-TAIL kit For Extreme 3′ RACE (Bioo Scientific) in accordance with the manufacturer’s instructions. In brief, an adenylated adaptor oligonucleotide was ligated to the 3′ ends of total RNAs by a T4 RNA ligase, and then reverse-transcribed using a primer specific to the adenylated adaptor. The cDNA was amplified by PCR with a primer designed near the 3′ end of the luciferase gene (5′-GATCCTCATAAAGGCCAAGAAGG-3′) and the primer specific to the adenylated adaptor. The PCR product was separated on an agarose gel.

#### Relative quantification of minigenomic RNA synthesis by real-time PCR.

Total RNA extracted from the minigenome assay described above was transcribed with PrimeScript reverse transcriptase (TaKaRa) using oligo(dT) primer for mRNA or trailer-specific primer for anti-minigenomic RNA (5′-ACCGAACAAGGGTAAAGAAG-3′). Comparisons of the expression levels of luciferase mRNA and anti-minigenomic RNA were performed by real-time PCR using SYBR Premix Ex *Taq* (TaKaRa) and a primer pair specific for the luciferase gene (5′-AATCCATCTTGCTCCAACACC-3′ and 5′-CGTCTTTCCGTGCTCCAA-3′). All data were normalized by the corresponding level of GAPDH mRNA using a specific primer pair (5′-CCACCCATGGCAAATTCCATGGA-3′ and 5′-TCTAGACGGCAGGTCAGGTCCACC-3′).

#### N–P–L complex formation assay.

pCAGGS-NiV-N (or N mutants), pCAGGS-NiV-P and pCAGGS-NiV-L were expressed individually in COS-7 cells using FuGene 6 transfection reagent, as described above. After lysis in 250 µl lysis buffer, an equal volume of cell lysate expressing N (or N mutants) was mixed with P and L, and incubated for 30 min at 4 °C. The supernatant of each sample was layered onto a discontinuous gradient containing 25, 30 and 40 % (w/w) caesium chloride and was then centrifuged as described above. The band material was collected and pelleted by ultracentrifugation. Each sample was subjected to SDS-PAGE and detected by Western blotting using antiserum against NiV-N, -P and -L, respectively.
